# Correlation Analysis of Suture Anchor Pull-Out Strength with Cortical Bone Thickness and Cancellous Bone Density on a Finite Element Model

**DOI:** 10.3390/bioengineering12080863

**Published:** 2025-08-11

**Authors:** Jung Ho Kim, Jeon Jong Hyeok, Jae Hyun Woo, Sung Min Kim

**Affiliations:** 1 Department of Regulatory Science for Medical Device, Dongguk University, Seoul 04620, Republic of Korea; jhkim1913@naver.com (J.H.K.); jjh30512@naver.com (J.J.H.); jaehyunwoo@dongguk.edu (J.H.W.); 2 Department of Medical and Healthcare, Dongguk University, Seoul 04620, Republic of Korea; 3 Department of Biomedical Engineering, College of Life Science and Biotechnology, Dongguk University, Seoul 04620, Republic of Korea

**Keywords:** suture anchor, cortical bone thickness, cancellous bone density, Finite Element Method (FEM)

## Abstract

This study aimed to assess, using finite element analysis (FEA), the mechanical effects of cortical bone thickness and cancellous bone density on the pull-out strength of suture anchors. A PEEK anchor was modeled and embedded in synthetic bone blocks with cortical thicknesses ranging from 1 to 5 mm and cancellous densities of 10 PCF, 20 PCF, and 30 PCF. Axial tensile loading simulations were conducted for all combinations, and selected cases were validated through experimental pull-out tests using commercial synthetic bone, demonstrating agreement within ±6%. Both cortical thickness and cancellous density were found to enhance pull-out resistance, though the magnitude and pattern varied with density. At 10 PCF, pull-out strength increased linearly with cortical thickness. At 20 PCF, substantial gains were observed between 2 and 4 mm, followed by a plateau. At 30 PCF, most of the increase was confined between 2 and 3 mm, with minimal improvement thereafter. These findings suggest that fixation strategies should be adapted on the basis of bone quality and provide biomechanical insights to inform patient-specific implant design and surgical planning.

## 1. Introduction

Soft-tissue injuries, including rotator cuff tears, are becoming increasingly prevalent in aging populations, and the use of suture anchors for arthroscopic repair has been established as the standard of care. The initial mechanical fixation provided by the suture anchor plays a critical role in tissue healing and reducing re-tear rates; therefore, comprehending the bone properties that influence anchor pull-out strength is crucial. Prior studies indicate that pull-out failures are relatively frequent and that prominent risk factors include bone quality, insertion depth and angle, the pattern of rotator cuff tear, and anchor design [1,2].

Bone demonstrates considerable variation in cortical thickness and cancellous density according to the anatomical site, which necessitates a patient-specific bone quality assessment before surgery when using suture anchors in these differing environments. For instance, radiographic investigations of the proximal humerus reveal mean cortical thicknesses of 2.34–5.24 mm, parameters that serve as critical markers for both fracture risk and implant stability [3,4], whereas the glenoid cortex is notably thinner (1.0–1.3 mm), presenting greater challenges for secure anchor fixation in this region [5]. In contrast, cortical thicknesses of 4.04–4.77 mm in the medial patella and 3.12–3.72 mm in the anterior inferior calcaneal cortex have been reported, emphasizing the demand for accurate regulation of insertion angle and depth at each anatomical location [6,7]. Furthermore, cancellous bone density decreases at a rate of approximately 1–2% annually with aging and is reduced by more than 30% in osteoporotic patients compared with healthy individuals, which is a leading factor contributing to diminished anchor pull-out strength [8].

While cortical thickness has historically been viewed as the chief determinant of anchor fixation strength, Ntalos et al. reported no statistically significant association between cortical thickness and pull-out strength for conventional screw-type anchors—as opposed to all-suture anchors—suggesting that other factors such as cancellous density, insertion technique, and anchor design or material substantially influence fixation outcomes [9]. Notably, Klein et al. applied in vitro tests using diverse screw configurations and revealed that reduced cancellous density markedly impairs pull-out resistance [10], while Tingart et al. established that local volumetric bone mineral density (vBMD) in cadaveric proximal humeri is directly related to anchor strength, underscoring both the detrimental effects of low bone density and the necessity for careful anchor selection in patients with inferior bone quality [11].

Recent FEA studies have demonstrated that incorporating detailed bone microarchitecture is critical for accurate pull-out predictions. For example, Ayoub et al. showed that varying the thread profile, tip geometry, and insertion angle in an FEA model markedly alters stress fields in both cortical and cancellous compartments [12]. Wang et al. correlated micro-CT-derived trabecular metrics (bone volume fraction, trabecular thickness) with measured pull-out forces in cadaveric humeri, underscoring the need for anatomically precise simulations [13]. Furthermore, Braunstein et al. found that cement augmentation in osteoporotic cadaveric bone significantly boosts pull-out strength across multiple anchor designs, highlighting how clinical augmentation strategies can be evaluated in silico before in vitro testing [14].

Despite this, the majority of current research has examined cortical and cancellous bone characteristics independently or has confined studies to biomechanical assessments of particular anchor shapes [15]. There is a clear lack of studies that systematically assess the pull-out strength of conventional anchors considering combinations of clinically relevant cortical thickness and cancellous density.

Therefore, the present study utilizes finite element analysis (FEA) in combination with focused in vitro validation to thoroughly investigate the pull-out characteristics of a PEEK screw-type anchor embedded within synthetic bone models featuring cortical thicknesses of 1–5 mm and cancellous densities of 10 PCF, 20 PCF, and 30 PCF [16]. The results are intended to establish bone quality thresholds for forecasting anchor fixation strength and to guide optimal decisions regarding insertion depth, angle, and anchor design, thereby supporting patient-specific surgical planning and advancement of implant design [17].

## 2. Methods

**Figure 1:**
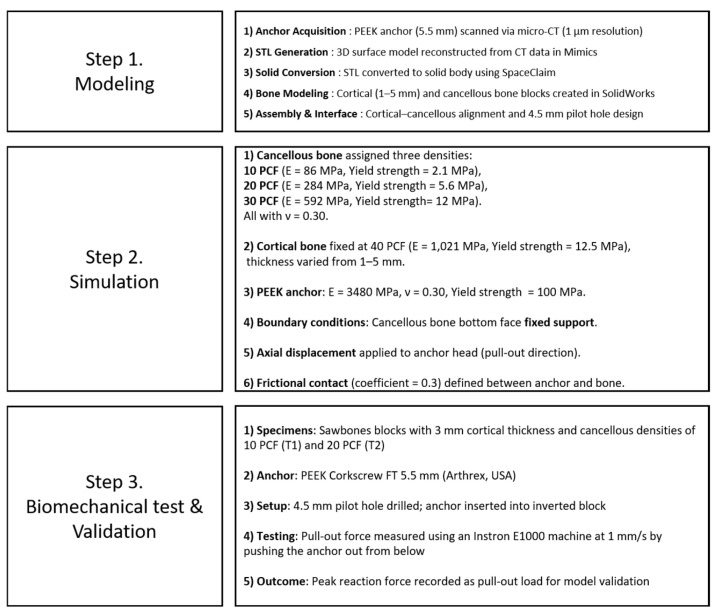
Overview of the methodological workflow comprising model construction, simulation setup, and biomechanical validation.

To investigate the effects of cortical bone thickness and cancellous bone density on the pull-out strength of suture anchors, this study employed a combination of finite element analysis (FEA) simulations and biomechanical validation tests. The overall methodological process was structured into three main steps: (1) modeling, (2) simulation, and (3) biomechanical testing and validation. Each stage is summarized in Figure 1.

### 2.1. Finite Element Modeling

**Figure 2:**
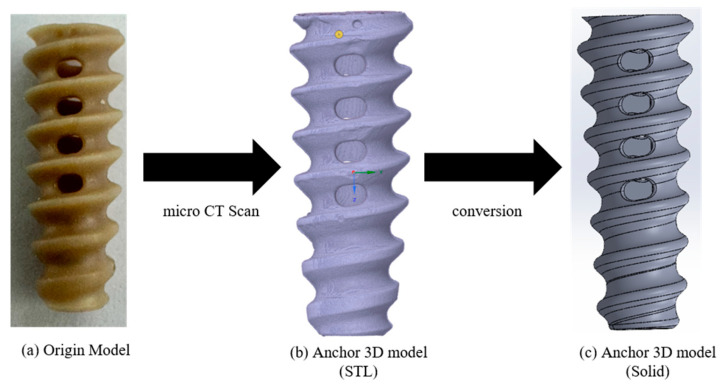
Workflow for reverse-engineering the suture anchor: (**a**) original PEEK Corkscrew FT anchor, (**b**) micro-CT-derived STL surface mesh, and (**c**) converted solid CAD model for FEA.

A commercially available suture anchor was selected to construct the FEA model for assessing pull-out loads in relation to cortical thickness and cancellous density. A PEEK Corkscrew FT 5.5 mm (Arthrex, Naples, FL, USA) was scanned using a micro-CT system (Vtomex m30 micro-CT (Bruker, Billerica, MA, USA); 1 μm resolution) to obtain DICOM image data. The DICOM images were then imported into Mimics (Materialise, Leuven, Belgium), where a 3D surface model in STL format was reconstructed. As the STL output from Mimics represents a hollow shell, it was converted to a fully solid 3D model using SpaceClaim (ANSYS, Inc., Canonsburg, PA, USA) (Figure 2).

**Figure 3:**
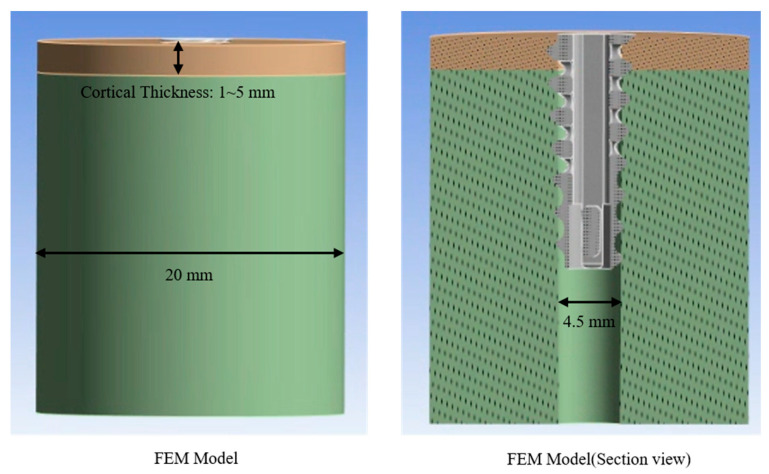
Finite element model of the synthetic bone block: (**left**) overall geometry showing the cortical layer thickness (1–5 mm) over a 20 mm cancellous core, and (**right**) sectional view illustrating the 4.5 mm pilot hole and inserted suture anchor.

The cortical and cancellous bone models for simulation were developed in SolidWorks 2023 (Dassault Systèmes, Vélizy-Villacoublay, France). To investigate pull-out loads under different cortical thicknesses, five cortical bone blocks were modeled with thicknesses of 1 mm, 2 mm, 3 mm, 4 mm, and 5 mm. The cancellous bone was simulated as a cylindrical block (radius = 10 mm). The Assembly tool in SolidWorks 2023 was used to ensure that the cortical and cancellous components were concentrically aligned. A pilot hole with a diameter of 4.5 mm—matching the inner diameter of the anchor—was introduced to define the contact interface between the synthetic bone assembly and the anchor (Figure 3).

The synthetic bone–anchor assembly used for simulating pull-out loads was constructed in SolidWorks 2023 (Dassault Systèmes, Vélizy-Villacoublay, France) via the Cavity feature: the PEEK suture anchor was inserted into the pilot hole’s surface, and any overlapping geometry was removed through subtraction. The assembled model was subsequently transferred to ANSYS R2023 (ANSYS, Inc., Canonsburg, PA, USA) for meshing and pull-out analysis. A 4-node tetrahedral mesh was constructed, assigning the cortical bone, cancellous bone, and suture anchor as distinct bodies. 

To ensure accurate stress distribution and interface behavior, an element size of 0.5 mm was applied to the anchor body and at the cortical–anchor and cancellous–anchor interfaces, while a 1.0 mm element size was assigned to the surrounding bone structures. This meshing strategy resulted in a total of 105,039 nodes and 70,485 elements, balancing computational efficiency with resolution. Although a formal mesh convergence study was not conducted in this investigation; previous studies involving pedicle screw models reported that using 0.6 mm elements for the implant and 1.0 mm for bone yielded pull-out force and stress predictions within 5% deviation compared with finer meshes. Therefore, the selected element sizes in this study are considered appropriate for capturing the global mechanical behavior of the bone–anchor system [18].

For comprehensive analysis, five models with cortical thicknesses of 1 mm, 2 mm, 3 mm, 4 mm, and 5 mm were generated, and the cancellous bone material properties were assigned densities of 10 PCF, 20 PCF, and 30 PCF to study the combined effects of cortical thickness and cancellous density (Table 1).

### 2.2. Material Properties and Boundary Conditions

Three distinct sets of material properties were assigned to cancellous bone, corresponding to densities of 10 PCF, 20 PCF, and 30 PCF, while cortical bone properties were fixed at the value for 40 PCF and only the thickness was varied. PCF (pounds per cubic foot) indicates the mass per unit volume, with higher PCF values signifying increased weight per volume and generally reflecting greater mechanical strength and stiffness. The specific properties for cancellous bone were as follows:

- 10 PCF: Density, 0.16 g/cm^3^; Young’s modulus, 86 MPa; Poisson’s ratio, 0.30; yield strength, 2.1 MPa;
- 20 PCF: Density, 0.32 g/cm^3^; Young’s modulus, 284 MPa; Poisson’s ratio, 0.30; yield strength, 5.6 MPa;
- 30 PCF: Density, 0.48 g/cm^3^; Young’s modulus, 592 MPa; Poisson’s ratio, 0.30; yield strength, 12 MPa.

A bilinear isotropic hardening model was applied to all bone materials to account for plastic deformation beyond the yield point, with yield strengths defined according to each density. The PEEK anchor was modeled with a density of 1.30 g/cm^3^, Young’s modulus of 3480 MPa, Poisson’s ratio of 0.30, and a yield strength of 100 MPa, based on values reported in biomedical literature [19,20]. The material properties for the polyurethane foam blocks were obtained from the manufacturer’s technical datasheet and the ASTM F1839 standard specification for rigid polyurethane foam used in orthopedic device testing [21,22] (Table 2).

A bonded contact was used to represent the interface between cortical and cancellous bone layers. This was implemented in ANSYS by assigning a bonded-type contact behavior to the internal interface, fully constraining all nodes along the shared surface to prevent any relative motion or separation. This setting reflects the monolithic structure of synthetic bone blocks in which both layers are manufactured as a continuous unit.

**Figure 4:**
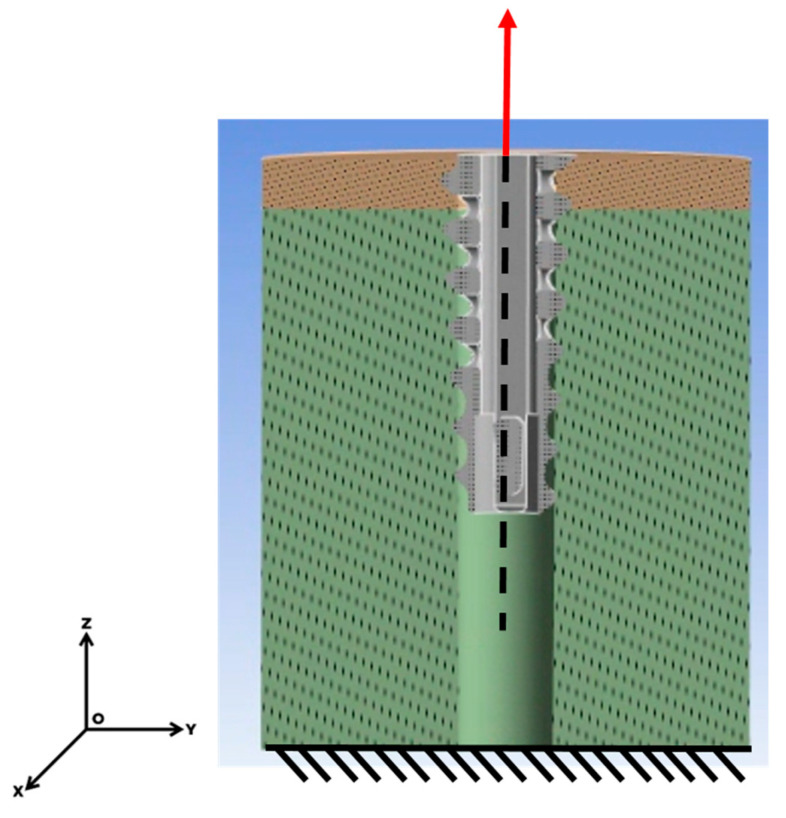
FEM boundary and loading setup.

The cortical–anchor and cancellous–anchor interfaces were treated as frictional contacts, employing a friction coefficient of 0.2 [23]. The base of the model was constrained using a fixed support, and displacement was applied to the anchor along the z-axis at a rate of 1 mm/s. The force response during anchor extraction was measured (Figure 4).

### 2.3. Push-Out Testing Method

The finite element model was validated by comparison with biomechanical test results obtained from a synthetic bone system (Sawbones, Pacific Research Laboratories, Vashon, WA, USA). 

Sawbones blocks were selected due to their standardized and reproducible mechanical properties, and have been shown to approximate the biomechanical behavior of human bone with reasonable accuracy in previous studies. For instance, Nagaraja and Palepu (2016) compared screw pull-out strength between Sawbones blocks and thoracolumbar cadaveric vertebrae, reporting differences within 5–15%, thereby supporting the suitability of Sawbones for model validation [24]. Among the 15 models analyzed, specimens Numbers 7 and 8 represented the commercial blocks, both with a cortical thickness of 3 mm and containing cancellous bone at 10 PCF and 20 PCF densities, respectively (Table 3).

**Figure 5:**
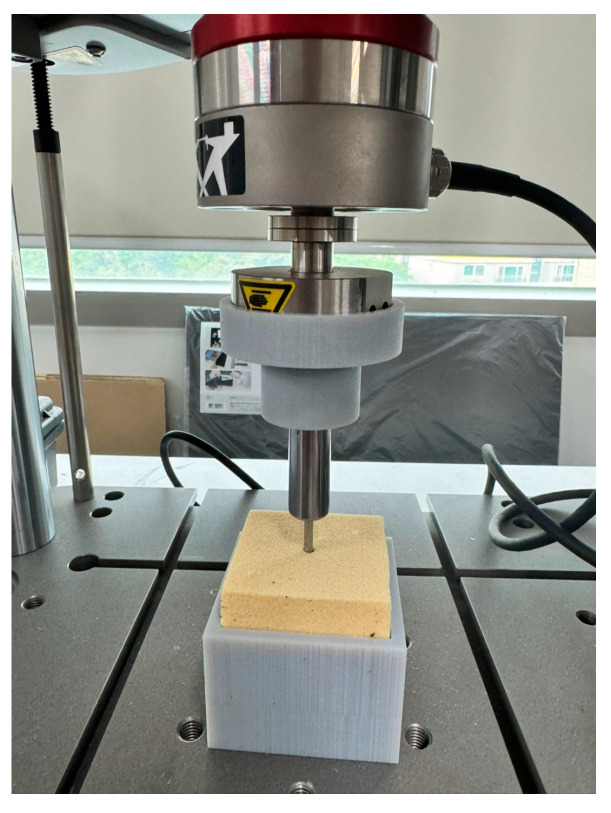
Push-out test setup.

For the Sawbones specimens with 3 mm cortical thickness and 10 PCF as well as 20 PCF cancellous densities, pilot holes measuring 4.5 mm in diameter were drilled to replicate the conditions of the FEA. The PEEK Corkscrew FT 5.5 mm anchor (Arthrex, Naples, FL, USA) was implanted, and pull-out loading tests were performed using an Instron E1000 universal testing machine (Instron, Norwood, MA, USA) at a displacement rate of 1 mm/s. Owing to the absence of a screw head on the anchor, which precludes securement with standard pull-out fixtures, each Sawbones block was inverted, and a push rod was applied to advance the anchor out of the block, with the generated reaction force recorded and interpreted as the pull-out load (Figure 5).

## 3. Results

### 3.1. Push-Out Test Result

**Figure 6:**
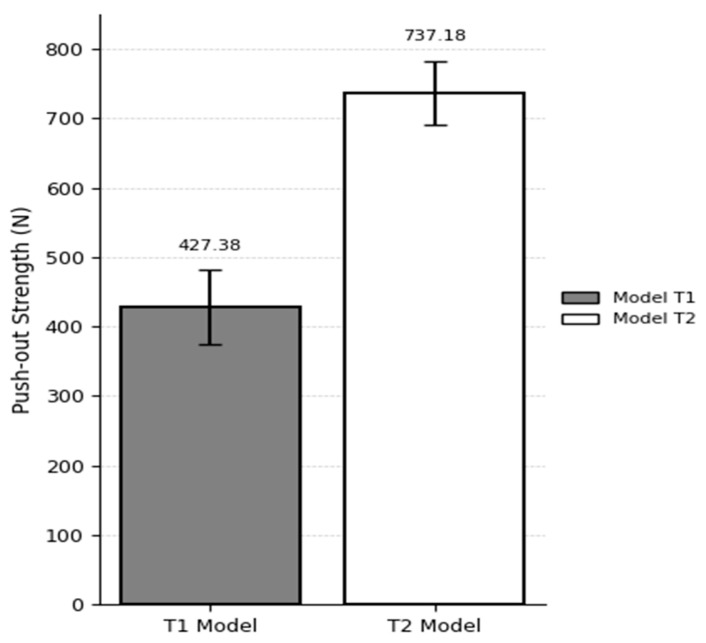
Comparison of pull-out loads for Models T1 and T2.

In the T1 model (10 PCF cancellous bone with a 40 PCF cortical layer and 3 mm cortical thickness), the suture anchor pull-out load was 427.38 ± 53.64 N, while in the T2 model (20 PCF cancellous bone with a 40 PCF cortical layer and 3 mm cortical thickness), the load reached 737.18 ± 45.78 N. These values were obtained from three repeated biomechanical tests per group. The 72.6% increase in pull-out strength with increasing cancellous bone density suggests that internal bone stiffness plays a critical role in enhancing the fixation strength of the anchor (Figure 6).

### 3.2. FEM Result

In the S1–S3 models, where cortical thickness was set at 1 mm and cancellous density increased sequentially from 10 PCF to 20 PCF and then to 30 PCF, the simulated pull-out loads were 314 N (10 PCF), 520 N (20 PCF), and 907 N (30 PCF). Pull-out load increased by 65.6% between S1 and S2, and by 74.2% between S2 and S3.

For the S4–S6 models with a cortical thickness of 2 mm, the pull-out loads measured were 367 N, 580 N, and 938 N for increasing cancellous densities. The pull-out load rose by 58.0% from S4 to S5 and by 61.7% from S5 to S6.

Within the S7–S9 group, which had a cortical thickness of 3 mm, pull-out loads of 417 N, 780 N, and 1621 N were recorded. The increase was 87.0% from S7 to S8 and 107.8% from S8 to S9.

In the S10–S12 models with a cortical thickness of 4 mm, the measured pull-out loads were 483 N, 1078 N, and 1683 N, respectively. The increment from S10 to S11 was 123.2%, while from S11 to S12, it was 56.11%.

In the S13–S15 series, using a cortical thickness of 5 mm, the obtained pull-out loads were 518 N, 1157 N, and 1733 N. The increases observed were 123.4% from S13 to S14 and 49.8% from S14 to S15.

For cortical thicknesses ranging from 1 mm to 3 mm, the increase in pull-out load was greater when the cancellous density was raised from 20 PCF to 30 PCF, as compared with the increase from 10 PCF to 20 PCF, whereas for 4–5 mm cortices, the most substantial gain was seen between 10 PCF and 20 PCF (Table 4).

Among the models with a cancellous bone density of 10 PCF, increasing the cortical thickness produced only slight increases in pull-out load: raising the thickness from 1 mm to 2 mm yielded a 16.9% rise; between 2 mm and 3 mm, a 13.6% increase; from 3 mm to 4 mm, a 15.8% gain; and from 4 mm to 5 mm, a 7.2% increment.

In models with 20 PCF cancellous bone, the incremental increases in pull-out load for thicknesses of 1 mm to 2 mm, 2 mm to 3 mm, 3 mm to 4 mm, and 4 mm to 5 mm were 11.5%, 34.5%, 38.2%, and 7.3%, respectively.

**Figure 7:**
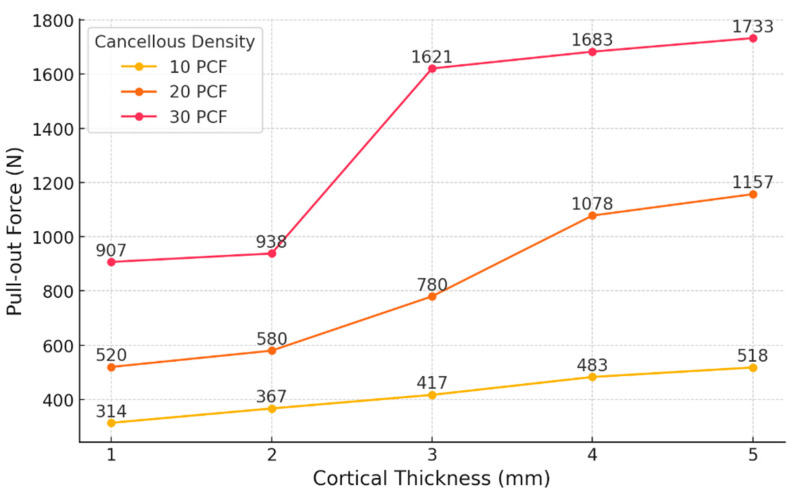
Influence of cortical thickness and cancellous bone density on anchor pull-out strength.

For the 30 PCF models, the pull-out load increased by 3.4% (from 1 mm to 2 mm), 72.8% (from 2 mm to 3 mm), 3.82% (from 3 mm to 4 mm), and 3.0% (from 4 mm to 5 mm) (Figure 7).

To quantitatively assess the influence of cortical thickness on pull-out performance, slope values (ΔForce/ΔThickness) were calculated between adjacent cortical thickness intervals for each cancellous density group (Table 5). For the 10 PCF models, the slope ranged from 35.0 N/mm to 66.0 N/mm, with the highest increase observed between 3 mm and 4 mm. In the 20 PCF group, the slopes varied from 60.0 N/mm (1–2 mm) to 298.0 N/mm (3–4 mm), indicating a more pronounced effect of cortical thickening in the mid-range.

Notably, the 30 PCF models exhibited a peak slope of 683.0 N/mm between 2 mm and 3 mm, while all other intervals showed minimal increases (<65 N/mm). These results indicate a nonlinear interaction between cortical thickness and cancellous density, with the most substantial gains occurring under moderate- to high-density conditions and mid-range cortical thickness (2–3 mm). 

### 3.3. Stress Distribution in Bone Under Screw Pull-Out Conditions

**Figure 8:**
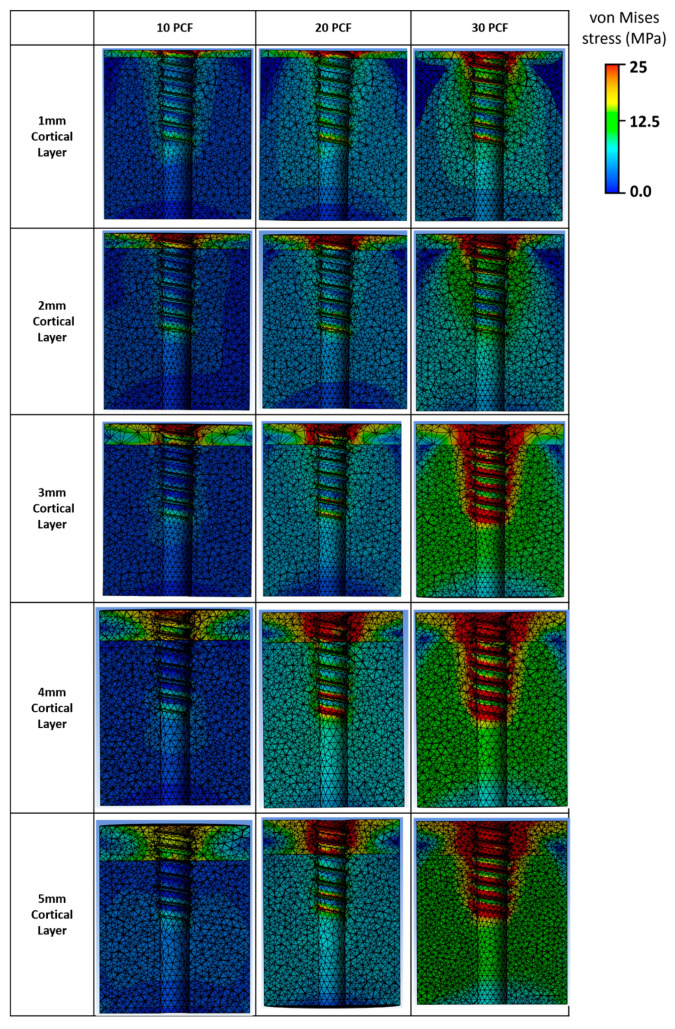
von Mises stress distribution for various cortical layer thicknesses (1–5 mm) and bone densities (10, 20, and 30 PCF).

Von Mises stress analyses were conducted to assess load transfer characteristics under varying cortical thicknesses and cancellous bone densities (Figure 8). The stress distribution patterns exhibited distinct differences depending on the mechanical properties of the surrounding bone structure.

Under conditions with low-density cancellous bone (10 PCF, yield strength: 2.1 MPa) and thin cortical layers (1–2 mm), stress was highly concentrated at the superior aspect of the anchor–cortex interface. The localized stress near the anchor tip rapidly reached the yield limit, resulting in confined plastic deformation regions around the anchoring point.

In contrast, when cancellous bone density increased to 20–30 PCF (yield strength: 5.6–12 MPa) and cortical thickness reached ≥3 mm (yield strength: 19 MPa), the stress distribution became broader and more uniform across the anchor–bone interface. The stress contours extended throughout both cortical and cancellous regions, indicating enhanced load sharing by the surrounding bone. High-stress areas were expanded in size but remained within the elastic range without localized concentration.

Overall, a transition was observed from highly localized stress peaks under weak bone conditions to widely distributed moderate stress under stronger bone configurations. These visual trends were consistent with the differences observed in measured pull-out forces across conditions.

### 3.4. Comparison of Experimental and Finite Element Analysis Model Results

To validate the simulation model, results from experimental pull-out tests were compared against predictions from finite element analysis (FEA). Test model T1 consisted of a synthetic bone block with a 3 mm cortical layer and a cancellous density of 10 PCF, in which the pull-out load of the PEEK suture anchor was evaluated. The corresponding simulation model S7 was constructed under matching conditions. The pull-out load for T1 was measured at 427.3 N, while S7 predicted a value of 417 N—indicating that the experimental result was 2.4% higher.

**Figure 9:**
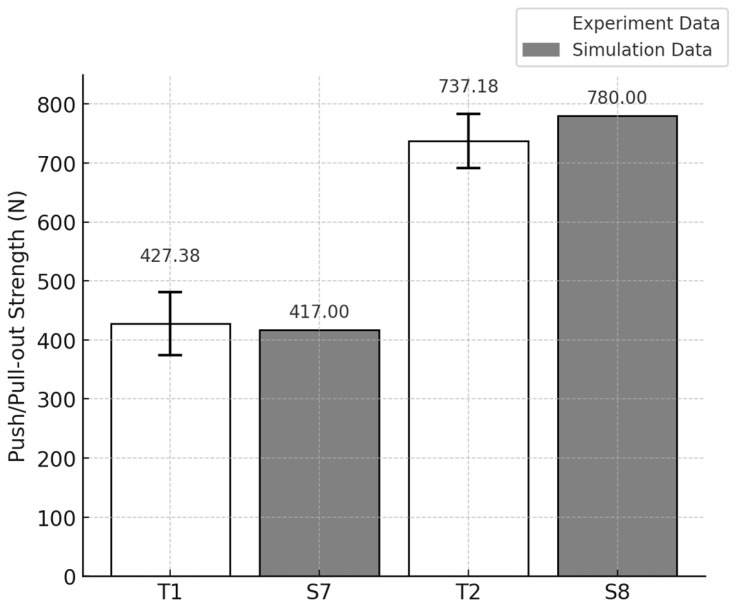
Comparison of experimental and finite element analysis model results.

For test model T2 (3 mm cortical layer, 20 PCF cancellous density), the experimental pull-out load was 737.18 N, and under these same conditions, the S8 simulation produced 780 N, which was 5.7% higher than the experimental measurement (Figure 9).

## 4. Discussion

In this investigation, finite element simulations of pull-out loads were conducted for orthopedic suture anchors across varying cortical bone thicknesses (1–5 mm) and cancellous bone densities (10 PCF, 20 PCF, and 30 PCF) to clarify their interdependent effects. Our FEA model was validated with a limited dataset (two experimental scenarios), and we quantitatively analyzed pull-out loads and their percentage increases as cortical thickness changed for each density condition.

### 4.1. Agreement Between Finite Element Model Predictions and Experimental Pull-Out Measurements

To evaluate model accuracy, experimental pull-out testing and FEA predictions were compared at cancellous bone densities of 10 PCF and 20 PCF, each paired with a 3 mm cortical layer. For the 10 PCF condition, the measured pull-out load was 427.38 N, and the FEA model predicted 417 N, yielding a 2.43% error. At 20 PCF density, experimental and simulated pull-out values were 737.8 N and 780 N, respectively, corresponding to a 5.72% difference. These findings demonstrate that the FEA model can replicate physical test results within ±6%, which is well beneath the generally accepted validation threshold of 10%. The particularly close agreement in the low-density (10 PCF) scenario supports the model’s validity in representing the structural behavior and bone–anchor interface characteristics during pull-out.

The opposite results observed between the two validation models may be explained by differences in the load transfer mechanisms associated with cancellous bone density. In the low-density T1 model (10 PCF), the experimental pull-out load may have been elevated due to micro-interlocking effects or localized inhomogeneity within the foam, which were not captured in the simulation.

In contrast, the higher pull-out load predicted by the simulation in the high-density T2 model (20 PCF) may be attributed to idealized assumptions regarding friction and contact behavior. Additionally, minor discrepancies in specimen fabrication, pilot hole fit, or insertion depth could have contributed to the reversed trend. Nevertheless, the differences between experimental and simulated results remained within the standard deviation of the measured data. These findings suggest that the finite element model maintains acceptable predictive accuracy, even across varying bone density conditions.

### 4.2. Simulation-Based Analysis of Pull-Out Load Variations with Cortical Thickness and Cancellous Bone Density

FEA model predictions indicated that pull-out load responses varied substantially in relation to cancellous bone density.

In low-density conditions (10 PCF), pull-out load exhibited a consistent and linear increase as cortical thickness ranged from 1 mm to 5 mm. The corresponding load increments were 16.9%, 13.6%, 15.8%, and 7.2%, maintaining a relatively uniform slope. These results demonstrate that when cancellous bone provides insufficient support, the cortical shell becomes the principal contributor to fixation strength, and progressive increases in cortical thickness reliably augment overall system stiffness.

In the mid-density groups (20 PCF), the effect of cortical thickening did not follow a linear pattern. The load increased by 11.5%, 34.5%, 38.2%, and 7.3% as cortical thickness rose from 1 mm to 5 mm. A moderate increase was observed from 1 mm to 2 mm (11.5%), whereas the most substantial gains occurred between 2 mm and 4 mm (34.5% and 38.2%), with a subsequent reduction to 7.3%. These findings indicate that at 20 PCF, the cortical layer provides its greatest contribution within the 2–4 mm thickness range, beyond which, additional thickening results in diminishing mechanical returns. Therefore, maintaining at least 4 mm of cortical thickness within medium-density cancellous bone may be optimal for improving pull-out strength.

In the high-density scenario (30 PCF), an even steeper pattern was observed. The load increased by just 3.4% from 1 mm to 2 mm, surged by 72.8% from 2 mm to 3 mm, and then dropped markedly to 3.8% and 2.97% for 3→4 mm and 4→5 mm, respectively. These results demonstrate that, for cortical thicknesses below 2 mm, changes have little influence on fixation; however, increasing to 3 mm leads to a pronounced improvement in pull-out resistance. Beyond this threshold, mechanical saturation limits the benefit of further thickening.

Although linear elastic material properties were used for all components, the pull-out force did not increase linearly with cortical thickness. This can be attributed to structural interactions rather than the material’s behavior. As cortical thickness increased, changes in local stiffness and load transfer between cortical and cancellous bone likely caused disproportionate gains in pull-out strength. At 30 PCF, the 72.8% increase from 2 mm to 3 mm suggests that the cortical layer began to play a dominant role in load resistance. In contrast, the 7.3% increase from 4 mm to 5 mm at 20 PCF may indicate a saturation point where additional thickness has limited effect. Frictional contact at the anchor–bone interface may also contribute to these nonlinear trends.

Slope analysis (ΔForce/ΔThickness) provided further insight into load transfer efficiency under varying bone conditions. In the 10 PCF models, the slopes remained relatively consistent (35.0–66.0 N/mm), suggesting that the cortical layer served as the primary load-bearing structure in the absence of sufficient trabecular support. For the 20 PCF group, the slopes increased sharply between 2 mm and 4 mm (up to 298.0 N/mm), indicating that cortical thickening becomes more effective when moderate cancellous density enables partial load sharing. Notably, in the 30 PCF condition, the slope peaked at 683.0 N/mm between 2 mm and 3 mm and declined thereafter, suggesting a structural synergy that maximizes load resistance within a specific thickness range, beyond which, mechanical saturation occurs [25].

### 4.3. Stress Distribution-Based Interpretation of Pull-Out Behavior

In this study, a bilinear isotropic hardening model was adopted to account for the nonlinear mechanical behavior of bone beyond the elastic range, with yield strengths assigned according to the density of the cancellous bone. This approach enabled an evaluation of not only the magnitude of stress but also the post-yield load transfer characteristics and their association with fixation performance.

Under the 10 PCF condition (yield strength: 2.1 MPa), localized stress near the anchor tip quickly reached the yield limit, resulting in early plastic deformation and a limited capacity for further stress accumulation. These patterns aligned with concentrated stress fields and lower pull-out forces, suggesting reduced fixation reliability.

In contrast, models with 20–30 PCF cancellous bone (yield strength: 5.6–12 MPa) exhibited more favorable stress distributions, with broader dispersion and greater structural participation in load transfer. The combination of thick cortical bone (≥3 mm) and high-density cancellous bone produced a more stable and distributed stress pattern, contributing to significantly improved fixation strength.

These results highlight a strong correlation between the mechanical strength of the bone substrate and the observed stress behavior. Notably, the distribution of stress rather than merely the peak stress values emerged as a key determinant of anchor performance. Visual analysis of von Mises stress maps provided a structural rationale that complements the quantitative pull-out data and reveals underlying load pathways and potential failure zones that are not readily apparent from force data alone [26].

Altogether, this study reinforces the importance of implementing yield strength-based material models to improve simulation accuracy and lays the foundation for future biomechanical investigations incorporating nonlinear behavior and patient-specific bone properties.

### 4.4. Clinical Implications of Pull-Out Load Analysis

Within low-density cancellous bone (10 PCF), pull-out resistance increased in direct proportion to cortical thickness, implying that thicker cortical shells are necessary for adequate anchor fixation. In the medium-density setting (20 PCF), the most prominent improvement in fixation was seen as cortical thickness increased from 2 mm to 4 mm. In high-density cancellous bone (30 PCF), the most significant rise in pull-out strength was observed between 2 mm and 3 mm of cortical thickness.

From a clinical perspective, these patterns support a tailored approach based on the characteristics of cancellous density and cortical thickness. For osteoporotic patients with low-density bone, maximizing cortical shell thickness consistently improves pull-out resistance. Patients with intermediate cancellous density appear to benefit most from ensuring a cortical shell of at least 4 mm. In young individuals with high-density cancellous bone, baseline fixation is robust, but a cortical thickness of 3 mm or greater produces the largest gain in pull-out strength.

### 4.5. Study Limitations and Future Research Directions

This investigation validated the finite element analysis (FEA) model by comparing simulated pull-out loads with experimental data collected under two specific conditions—cancellous bone densities of 10 PCF and 20 PCF, each paired with a 3 mm cortical layer. On the basis of this limited validation, the study further quantified the effects of cancellous density and cortical thickness on pull-out strength and its rate of change. While the simulation results demonstrated a high level of accuracy within ±6%, additional biomechanical tests covering a broader range of density and thickness parameters are necessary to enhance generalizability.

Although a bilinear isotropic hardening model was applied to account for post-yield plasticity, both cortical and cancellous bone were simplified as homogeneous and isotropic materials. In reality, bone exhibits complex anisotropic and nonlinear behaviors depending on the anatomical region and loading direction. This modeling assumption, while enhancing computational feasibility, may reduce the physiological accuracy of the results.

Moreover, the experimental validation was conducted using synthetic bone blocks (Sawbones), which differ from human bone in microarchitecture and biological heterogeneity. Although artificial bone allows for consistent and reproducible testing, it may affect the direct translatability of the findings to clinical applications.

To address these limitations, future work will incorporate nonlinear material models for cortical and cancellous bone and expand the experimental matrix to include a wider range of bone densities and cortical thicknesses. In addition, by leveraging the load trends identified in this study, we aim to optimize anchor design through geometric and material modifications to achieve site-specific configurations tailored to anatomical variation.

## 5. Conclusions

This study examined the pull-out resistance of orthopedic suture anchors in relation to cancellous bone density (10 PCF, 20 PCF, and 30 PCF) and cortical bone thickness (1–5 mm) using both experimental testing and finite element analysis (FEA). The simulation results enabled a detailed characterization of the influence of cortical thickness on fixation strength and quantification of the changes in pull-out load and its percentage increase across different density conditions.

For low-density cancellous bone (10 PCF), pull-out load displayed a steady linear increase with cortical thickness, demonstrating that increased cortical thickness consistently strengthens anchor fixation when cancellous support is weak. In medium-density bone (20 PCF), the most significant improvement in pull-out load was observed between 2 mm and 4 mm of cortical thickness, with minimal benefit beyond 4 mm, indicating that the 2–4 mm range is mechanically optimal under these circumstances. For high-density cancellous bone (30 PCF), a pronounced increase in pull-out load occurred only between 2 mm and 3 mm of cortical thickness, with reduced gains outside this interval, suggesting that cortical reinforcement achieves maximum efficacy after surpassing a critical threshold.

These results show that the mechanical role of cortical thickness varies—either linearly or nonlinearly—depending on the cancellous density and highlight that optimal cortical thickness for achieving maximum fixation should be determined by bone quality. The findings underscore the value of FEA-based load analysis in preoperative fixation planning, implant design refinement, and patient-specific surgical decision-making. Future research will extend model validation with additional biomechanical assessments and integrate nonlinear bone material behaviors to enhance clinical applicability.

## Figures and Tables

**Table 1:** Summary of finite element model configurations detailing cortical layer thickness and cancellous bone density for each model type.

Model Type	Cortical Layer Thickness	Cortical Layer	Cancellous Layer
S1	1 mm	40 PCF	10 PCF
S2	1 mm		20 PCF
S3	1 mm		30 PCF
S4	2 mm		10 PCF
S5	2 mm		20 PCF
S6	2 mm		30 PCF
S7	3 mm		10 PCF
S8	3 mm		20 PCF
S9	3 mm		30 PCF
S10	4 mm		10 PCF
S11	4 mm		20 PCF
S12	4 mm		30 PCF
S13	5 mm		10 PCF
S14	5 mm		20 PCF
S15	5 mm		30 PCF

**Table 2:** Mechanical properties of cancellous foam (10–40 PCF) and PEEK anchor material used in finite element simulations.

PCF	Density (g/cm^3^)	Young’s Modulus (MPa)	Poisson’s Ratio	Yield Strength (MPa)
10 PCF	0.16	86	0.3	2.1
20 PCF	0.32	284	0.3	5.6
30 PCF	0.48	592	0.3	12
40 PCF	0.64	1000	0.3	19
PEEK	1.3	3480	0.3	100

**Table 3:** Validation models: test conditions and corresponding FEM models.

Test Model	Cortical PCF	Cancellous PCF	Cortical Thickness	Validated Finite Element Model
T1	40 PCF	10 PCF	3 mm	S7
T2	40 PCF	20 PCF	3 mm	S8

**Table 4:** Simulated pull-out loads by cortical thickness and cancellous bone density.

Model Category	Cortical Thickness	Cancellous Bone Density	Simulation Outcome (N)
S1	1 mm	10 PCF	314
S2		20 PCF	520
S3		30 PCF	907
S4	2 mm	10 PCF	367
S5		20 PCF	580
S6		30 PCF	938
S7	3 mm	10 PCF	417
S8		20 PCF	780
S9		30 PCF	1621
S10	4 mm	10 PCF	483
S11		20 PCF	1078
S12		30 PCF	1683
S13	5 mm	10 PCF	518
S14		20 PCF	1157
S15		30 PCF	1733

**Table 5:** Slope of the increase in pull-out force (N/mm) across cortical thickness intervals at varying cancellous bone densities.

Cortical Thickness Interval (mm)	10 PCF (N/mm)	20 PCF (N/mm)	30 PCF (N/mm)
1–2	53.0	60.0	31.0
2–3	50.0	200	683.0
3–4	66.0	298.0	62.0
4–5	35.0	79.0	50.0

## Data Availability

The datasets supporting the conclusions of this study can be obtained from the corresponding author upon reasonable request.
